# Metal-Organic Frameworks (MOFs) Based Heterojunction for the Photocatalytic Degradation of PFAS

**DOI:** 10.1063/4.0001143

**Published:** 2025-10-27

**Authors:** Adarsh Nayarassery Narayanan

**Affiliations:** University of Texas at Dallas, Richardson, TX, USA

## Abstract

Per- and polyfluoroalkyl substances (PFAS) are persistent environmental contaminants found in groundwater, surface water, soil, and air, with prolonged exposure linked to serious health risks. To mitigate this growing concern, various PFAS removal strategies have been investigated, including anion exchange resins, adsorption using powdered or granular activated carbon, and redox-based degradation via photochemical, sonochemical, or electrochemical methods. Among these, photocatalytic degradation using sustainable materials has garnered significant interest due to its low environmental impact and potential for complete mineralization of PFAS. In this study, we report the development and application of a heterojunction photocatalyst composed of carbon nitride (C3N4) and UiO-67(Ce), a cerium-based metal-organic framework (MOF). This composite leverages the high adsorption capacity of UiO-67(Ce) for both PFSAs and PFCAs, coupled with its and C3N4’s photocatalytic properties to enhance PFAS degradation. We present the first fundamental findings from this research, highlighting the material's performance and mechanistic insights into its photocatalytic activity.

